# “We’re all in the same storm, but not all of us are in the same boat”: qualitative exploration of UK response-focused civil servants experiences of working from home during COVID-19

**DOI:** 10.1186/s12889-025-21385-4

**Published:** 2025-01-23

**Authors:** Charlotte E. Hall, Samantha K. Brooks, Neil Greenberg, Dale Weston

**Affiliations:** 1 Department of Psychological Medicine, King’s College London, Weston Education Centre, London, UK; 2 Behavioural Science and Insights Unit, Evaluation & Translation Directorate, Science Group, UK Health Security Agency, Porton Down, Salisbury, UK

**Keywords:** Work from home, Civil service, UK Government, COVID-19, Homeworking, Qualitative, Recommendations, Wellbeing

## Abstract

**Introduction:**

The experiences of UK Government response-focused employees, who were considered frontline workers during the coronavirus response, are missing from current literature. Meeting the demands of being on the frontline, whilst also adjusting from a normal and practiced way of working to having to work from within one’s home, may bring a plethora of new barriers and facilitators associated with providing an effective pandemic response.

**Method:**

This interview study collected and analysed data from 30 UK Civil servants who worked on the COVID-19 pandemic response from their own homes. Interviews aimed to: (1) explore UK Government employee’s experiences of working from home whilst contributing to the pandemic response; and, (2) establish what support and guidance employees were offered, and what they would recommend for future public health emergencies requiring homeworking.

**Results:**

Seven themes were extracted from the data: overall experience of working from home; preparedness for working from home; experience of contributing to the response effort; work life balance; relationships with colleagues; space and equipment; and, inclusivity. Findings suggested that during the pandemic, participants reported feeling a strong sense of purpose and achievement for contributing to the response. But, the work was demanding, particularly for those who had to rapidly, and unexpectedly, transition from office or lab work to home working. More generally, the nature of their homeworking experience depended on a range of practical (e.g., space in the home), organisational (e.g., relationships with managers) and personal factors (e.g., caring responsibilities). Many participants were underprepared to work from home, but participants provided a plethora of information relating to what support offers they would find useful during future work on the frontline from their own homes.

**Conclusion:**

The results of this study demonstrate that frontline UK Civil servants may need more tailored and flexible multilevel support (i.e., from peers, managers, organisations) during future public health emergencies when they are required to work from home. A series of data-informed recommendations are created and discussed.

**Supplementary Information:**

The online version contains supplementary material available at 10.1186/s12889-025-21385-4.

## Background

Due to the spread of coronavirus (COVID-19), in March of 2020 the World Health Organisation declared a global pandemic [1]. In the United Kingdom (UK), many behavioural interventions were implemented to aid in reducing transmission of the virus – which included those in employment being asked to “work from home if you can” in March of 2020 [2]. As of the following month, half of those in employment in the UK were reported to work from home [3],

The published literature on the effects of working from home is often mixed, for example stating some people prefer home working due to eliminating commuting time [4], whereas others struggle with blurred boundaries between work and home life [5]. Working from home has also been reported to have a mixed impact on both, mental health and productivity: the relationship is reported to vary considerably, suggesting a complex association, with many confounding factors (e.g., demographics, occupation, responsibilities; [6]). Due to the variability apparent across literature, and broad findings from a recent umbrella review focusing on the experiences of working from home [7], focusing on exploring experiences of specific occupational groups may aid in understanding and improving the working from home experience [7].

The experiences of various occupations working on the frontline during the COVID-19 pandemic are well documented amongst current literature (e.g., hospital workers [8, 9], teachers [10, 11], social workers [12]). Aside from healthcare workers, exploration into the experiences of the remaining essential workers during the COVID-19 pandemic is limited. For example, such is the case for local and national civil servants (also known as civil servants). In line with government restrictions, as of the 16th of March 2020, civil servants in the UK were required to work from home, including those actively contributing to, and providing, effective delivery of the coronavirus response [13]. The COVID-19 pandemic also altered the broad objectives of ministries and agencies entirely [14, 15]. This resulted in changes to structure, redeployment of employees (i.e., secondments), and the general mechanisms of funding sources changing (i.e., transfer of work from ‘business as usual’ to COVID-19 focused working; [14, 15]). COVID-19 thus fundamentally changed where and how civil servants work, the demands placed on them, and the demands they faced outside their role [14]. Meeting the demands of being on the frontline, whilst also adjusting from a structured and practiced way of working to having to work from within one’s home, may bring a plethora of new barriers and facilitators to providing effective delivery of the COVID-19 response.

Local and national civil servants have been included in research at a higher level, for example in a two wave longitudinal studies which sought to establish the mental health and wellbeing of essential workers in the UK [16]. Data were analysed across two monthly waves (March 2020, April 2020) seeking to compare frontline workers with the rest of the UK population on anxiety, depression, and PTSD prevalence. Blanketly, frontline workers were found to experience higher levels of depression, anxiety, and PTSD in comparison to non-frontline workers [16]. Local and national civil servants did not differ from non-frontline workers in mental health outcomes in the first week of lockdown but were reported to experience significantly higher levels across all assessed mental health measures one month later (in the second wave; [16]. In fact, PTSD was thought to be the primary mental health problem experienced by frontline workers, and was most prominent amongst government workers, public service workers, utility workers and public safety workers [16]. Government workers were also the only group to experience a significant increase in depression prevalence between waves; no other significant values across mental health status were found for any other frontline worker group [16]. Additionally, the Annual Civil Service People Survey (which ran between 2012 and 2022 [17]) demonstrated a vast decrease in life satisfaction, happiness, anxiousness, and belief that things in life are worthwhile for civil servants in 2020, which coincides with the COVID-19 pandemic and the working from home instruction.

Given the suggested impact to mental health and wellbeing, the novel context of working from home whilst working on the frontline COVID-19 pandemic response has not yet been explored within current homeworking literature. Given this, the current study aimed to: (1) qualitatively explore frontline UK Government employee’s experiences of working from home whilst contributing to the COVID-19 pandemic response, in the form of initial perceptions, barriers, facilitators, reflections and lessons learnt; (2) establish what support and guidance employees were offered, and what they would recommend for future public health emergencies requiring homeworking. Understanding employee experience will aid in identifying and, exploring the impact of, factors that play a key role in positive/negative experiences of working from home whilst on the frontline, and how they relate to mental health and wellbeing. These findings will be synthesised, and key evidence-informed recommendations will be derived.

## Method

### Design

The current study involved the conduct of interviews with UK Civil servants who worked on the frontline during the COVID-9 pandemic. A total of 30 interviews were carried out, with the aim of sufficiently reaching data saturation [18]). A semi-structured method was chosen to allow the interviewer freedom to ask for elaboration on particular answers, whilst also allowing the participant to direct the flow of the interview [19]. The COnsolidated criteria for REporting Qualitative research (COREQ) Checklist [20] was followed to allow comprehensive and thorough reporting of the current qualitative research study and is provided in the supplementary files.

### Participants and recruitment

To be eligible for inclusion, participants had to be over the age of 18 and have experience of working for the UK government on the COVID-19 response whilst working from home. All employees recruited were from the same government organisation. Participants were recruited via gatekeepers (i.e., UK Government team and department leads, or equivalent). To reach current employees, a short summary of the study, as well as a sign-up link, was distributed organisation wide using a weekly newsletter (which can be found in the supplementary files).

It was also decided to include previous employees in the interviews, where possible, to aid in overcoming bias in the sample (i.e., that some individuals who had been working on the COVID-19 response may have left the organisation, potentially due to if they had negative experiences, akin to the healthy worker effect (45)). To reach ex-employees, a small number of UK Government team and department leads were asked to distribute the same information (i.e., brief summary and link) to individuals they thought would meet the inclusion criteria.

### Procedure

The sign-up link directed participants to fill in some brief demographic and occupational information as a screening questionnaire, as well as leaving a preferred email address for contact. Potential participants were thanked for their interest, made aware that a total of 30 interviews were being conducted and, if selected, the first author would be in contact. A copy of the screening questionnaire can be found in the supplementary files.

The screening survey was completed 127 times, with meeting the inclusion criteria and choosing to leave a contact email address narrowing down the potential sample to 82. To ensure a broad range of disciplines (and in essence participant experiences) were included in the sample, the screening questionnaire asked participants which area they currently worked in within the organisation, and the area they worked in during the pandemic. The research team then used the organisational structure to randomly sample from across as many parts of the organisation as possible, and then used the nature of the potential participants’ COVID-19 work area to ensure that there was a broad range of experiences represented by the participants.

Selected participants were contacted by the first author and sent a consent form and participant information sheet. Due to non-responders, a total of 40 individuals were contacted about participating in the interviews, with 30 returning written consent and taking part. Interviews were scheduled upon the return of consent. All interviews were conducted by the first author between the 19th of August and the 19th of September 2023 and were held on Microsoft Teams. Following a completed interview, the participant was sent a £25 incentive voucher. Participants were not asked to review their transcripts, but were able to opt in to receive information about research findings and outcomes of the interview study.

### Data collection

The interviews began with the interviewer stating the purpose of this research, ensuring the participant did not mind being recorded, and asking if they had any questions prior to starting. In all cases, there were no other parties present, only the participant and first author (as interviewer). Once the recording began, the interview schedule was followed (please see the supplementary files). The interview schedule was originally informed by a previous review of literature conducted by the research team [7]. The interview schedule was piloted twice, informally, with an individual who met the inclusion criteria. The schedule was then developed and iterated post-pilot by the research team.

Interview topics included: (1) experience of initial transition to homeworking as a response-based Civil Servant during the COVID pandemic (e.g., *“Please could you tell me how you first found out that you would be working from home?*”); (2) experiences after working from home on the COVID response after a few months (e.g., “*Could you tell me about your experience after a few months of homeworking in comparison to the initial period?*”); (3) lessons learnt whilst working during the pandemic, and reflection on experiences (e.g., “*Could you briefly summarise your experience of working during the pandemic?*”); and, (4) based on personal experience, what support would have been useful when rapidly transitioning to homeworking whilst responding to a public health emergency. Interviews lasted between 27 min 04 s and 59 min 35 s (median 41 min 46 s); no repeat interviews took place.

Interviews were recorded and transcribed by Microsoft Teams. During the interview, the interviewer took brief notes of key topics and statements. Following each interview, the first author listened back to the recording and checked the transcript, amending all errors and transcribing verbatim where required (e.g., due to muffled talking, incorrect transcription).

### Data analysis

NVivo was used to store and code transcripts. The six-stage approach to reflexive thematic analysis was followed by the first author to analyse the data [21, 22]. Step one, familiarization of the data took place alongside writing notes during the interview, taking time after to reflect on the interviews, and reading through transcripts. The second step consisted of generating initial codes. The process was carried out line-by-line and consisted of assigning brief, but sufficiently detailed, codes to data excerpts. The third phase involved combining codes into themes and the fourth involved reviewing potential themes to ensure the themes represented the data accurately (e.g., ensuring there is enough meaningful data to support themes). The fifth phase consisted of defining and naming themes, and the sixth phase was to produce the report and select illustrative quotes to support themes. The research team provided feedback on the list of themes and chosen quotes to ensure they too thought they were representative of each theme and that the structure was logical.

### Ethics

The current study was carried out in accordance with the British Psychological Society Code of Ethics and Conduct [23], and was approved by the Psychiatry, Nursing and Midwifery Research Ethics Subcommittee at King’s College London (ethical clearance reference number: HR/DP-22/23-34829). Gatekeepers were used to recruit participants, to mitigate any pressure that may be felt by potential participants, they were assured that the gatekeeper would not know which staff members took part in the survey. Furthermore, the participant information sheet and screening questionnaire both stated that nobody from the UK Government organisation would know if they participated in the study or not. Individuals were also made aware that their participation was voluntary, and their data would be anonymised in any reporting. As a result, pseudonyms are used in reporting results. Informed consent to participate was obtained from all of the participants in the study.

### Reflexivity

Due to the qualitative nature of this research, the authors wanted to provide transparency in their roles and experience to allow the reader to consider the validity of the analysis. All interviews were carried out by the first author (CEH), a female PhD Student, who has been trained in, and has prior experience of, conducting qualitative data collection. All authors of the current article have experience of contributing to the COVID-19 response from their own homes.

## Results

30 participants were interviewed. In summary, ages ranged from 27 to 57 years (mode: 44) and 80% of participants were female. Number of people living in the household ranged from one to five (mode: 3), and ~ 50% of participants (*n* = 16) had children in the household. In terms of occupational characteristics, ~ 50% of the sample (*n* = 14) joined the organisation during the COVID-19 pandemic, with 50% having experience response-based work before the pandemic. 14 participants had some experience of working from home prior to the pandemic, which ranged from ‘minimal’ (i.e., the odd one or two days) to full-time homeworkers. Lastly, at the time of interview, six interviewees (i.e., 20%) no longer worked for the organisation.

One participant was asked to work from home earlier than others due to concerns about them catching COVID-19 as they were living with a chronic illness; one participant began working from home earlier than others due to health treatments; and two participants wished to work from home due to chronic conditions and mobility issues.

A total of seven themes were extracted from the data: overall experience of working from home; preparedness for working from home; experience of contributing to the response effort; work life balance; relationships with colleagues; space and equipment; and, inclusivity. Table 1 details all themes and subthemes.

**Table 1:** Themes and sub-themes

Theme	Subtheme(s)
Overall experience of working from home	Initial reactions to working from home; Workplace support provided
Preparedness for working from home	Participants suggestions to support preparedness
Experience of contributing to the response effort	Privilege; Workplace demands; External support; Moving and taking breaks; Participants suggestions for wellbeing support
Work life balance	Convenience; Household calling; Blurred boundaries; Participants suggestions for aiding positive work life balance
Relationships with colleagues	Participants suggestions for maintaining social connectedness
Space and equipment	Participants suggestions for space and equipment support
Inclusivity	Ways of working preference; Participants suggestions for supporting workplace changes

### Overall experience of working from home

When asked about their overall experience of working from home on the pandemic response during the COVID-19 pandemic, participants’ responses were mixed. Some were positive: *“I loved working from home. I feel like I’m still such like a home working person” (Helen)* and some were negative: *“the word that is in the front of my head is stressful*,* [.] my life was all encompassed around work” (Peter)*. Many acknowledged that there were both benefits and challenges: *“I couldn’t say it was good or bad*,* I think. It definitely had its perks*,* but it also definitely had its negatives for sure” (Amy)*. Additionally, some reported that the experience became better over time (i.e., as people started to settle into the change): *“it was initially sort of unsettling to begin with*,* and then later on everything sort of worked smoothly and lessons learned were basically how to cope in a situation whereby you not in your normal office setting” (Steve)*.

### Initial reactions to working from home

Participants’ initial feelings about working from home varied. Some found the news came as a shock: *“that decision came in really*,* really quickly*,* I don’t think we were expecting it”* (Peter), with others referring to the transition as chaotic or hectic, suggesting that it caused a degree of worry: *“State of panic*,* anxiety and sort of how long would this be for? How am I going to work and look after my daughter? How are three of us going to work at home? What about all my lab stuff?”* (Heather).

Participants also believed that the change to working from home would not last long, so initially it did not cause too much impact. Furthermore, some found the change to be irrelevant as the demands of work meant that there was not much time to feel concerned about ways of working and as everyone was in a similar position, it seemed manageable: *“There was very little time to even think about*,* you know*,* the computer screens or computer screen*,* whether it’s in the office or not*,* and the pressure of work in that period was extreme” (Daniel).*

As for new starters that joined during the pandemic, many felt happy to have a new role as well as grateful for the opportunity to work during the pandemic, but the nature of the response work (i.e., hectic, high demands and quick pace) meant that some were nervous and found initial integration into online teams a bit daunting: *“It was definitely a little bit awkward. I think*,* you know*,* I didn’t meet a single one of my colleagues for*,* like*,* nearly eight months” (Lauren)*. One participant remarked they did not mind only meeting colleagues online and attributed that to being younger: *“Maybe because I was younger*,* I was like 24. So*,* I feel like I was used to kind of meeting people online and like talking to people online anyway” (Helen)*.

### Workplace support provided

Common examples of support provided by the organisation during pandemic working from home, were referred to as “*the standard lines of the wellbeing support offer” (Peter*) with specific mentions for: *“apps like Calm” (Cara)*; *“wellbeing newsletters” (Cara)*; *“tips and tricks” (Amy)*; *“regular emails with what was available for support” (Mary)*; *“staff support networks” (Paul)*; and, *“a counselling team*, [called the] *EAP helpline” (Jane)*. Intranet and civil service-learning platforms were also mentioned (this was particularly true for new starters who joined during the pandemic): *“there was stuff on the Internet that was really useful” (Paul)*.

Across the organisation, support offers seemed to vary; some recalled many different support opportunities in their particular teams and networks. Some examples consisted of: *“Team wellbeing talks” (Cara)*; *“huddles” (Bethany)*; *“Chair yoga” (Holly)*; *“Coffee roulette” (Holly)*; *“[senior staff being] always available and accessible” (Emily)*, and a general *“encouragement to take time away from your desk and log off at the end of the day” (Cara)*.

Similarly, managers were frequently mentioned by many of the participants in response to workplace support at the start of the pandemic. This working relationship seemed to heavily impact the working experience. For most, participants reported healthy relationships with their managers, with many stepping up to provide more support when necessary, which was appreciated by participants: *“my line manager was very good and was highly supportive*,* I recognised that that wasn’t a formal organisational offering […]*,* it was only my line manager who had responsibility for a fair portion of the team who kind of took it upon himself to try and to tackle that wellbeing piece” (Peter)*. However, a few participants recall wishing for more support from their managers: *“my immediate line manager was not supportive*,* well*,* she was supportive*,* but she kind of abandoned me […] there was never any kind of one-to-one support” (Heather)*.

It is important to note, that some participants were unable to recall formal offerings of support at the start of the working from home initiative, with some suggesting that there was no time to consider anything other than the pandemic response: *“I honestly have no remembrance of anything*,* that’s not to say that something wasn’t sent. But we*,* you know*,* I have to say I had nose to the grindstone*,* head down in that period” (Daniel)*. Although, there was the general suggestion that more mental health and wellbeing resources were released over time, participants reflected that these should have been prioritised sooner: “*We did start to get a lot more sort of like support and stuff*,* probably more towards the end of the pandemic. So*,* I think that those sorts of things would be good to sort of like be implemented maybe a bit sooner” (Louise)*.

### Preparedness for working from home

Many participants reported feeling prepared to work from home for many reasons including: they had all the required equipment; had enough space in the home to work in a separate environment to the rest of the household; having previous experience of working from home; they expected to be working from home when joining the organisation; the presence of good communication channels for colleagues; and having prior experience of working alone. Some participants believed that they were partly prepared, but making the change was difficult and took time: *“I suppose the concept of working from home didn’t feel like*,* how the hell am I getting to do this? I think it was more the concept of*,* I’ve got to work here for an indefinite amount of time*,* and the reality was I didn’t have the right kit. I didn’t have the volume of equipment that I ended up having a needed to have” (Peter)*.

A few participants felt completely unprepared to work from home; it is necessary to note that all these participants were working for the organisation when the pandemic was declared and were not expecting to need to work from home at all: *“No*,* not at first. Equipment wise*,* you know the DSE* [display screen equipment] *wasn’t that great so I had to work around that*,* I was mainly working at the kitchen table. Also […] when you first start working at home*,* it’s quite difficult to […] know what to get on with first” (Emma)*.

### Participants suggestions to support preparedness

There was a focus from many participants on the importance of being able to provide tailored support to employees: “*Everyone needs their individual bit of support*,* which is going to be slightly different because you know […] if I had like 3 kids*,* I’d have a totally different experience (Jack)*”. Similarly, one participant described the experience as: *“we’re all in the same storm*,* but not all of us are in the same boat”* (Josh). Being considerate and tailoring support to meet different requirements, such as pre-existing mental health conditions and physical disabilities was also mentioned. Wider suggestions to tailor support included: the context in which working from home is introduced, “*if you’re not expecting it. Well*,* you’re not prepared. (Mary)*”; and for new starters: *“I think if you’ve got new people coming into the organisation […] depending on what their previous experience has been around being online [.] and how to use things like teams and Skype.” (Louise).*

Many participants suggested an accessible resource containing available guidance and support both for wellbeing and workplace guidance would be helpful: *“Knowing that they’ve [employees] got everything there in place and they don’t need to really worry about*,* what*,* what would I do if?” (Steve).* Essentially, “h*aving all the information […] is key. Making sure that the top priority is health and wellbeing*,* so keep that at the forefront*,* and then of course have all the information they [employees] need to be able to work.” (Steve)*.

There were many suggestions as to what could be included in a support package for employees. For example: all “*meetings and interactions that are available” (Josh);* “*having a checklist of things that they [employees] need to have access to*” (Josh); “*basic sort of technology”* aids (Steve); *“instructions on how [.] to get themselves set up [.] to work from home and what they are expected to do” (Steve)*; details of *“all the support that is provided by the organisation”* (Steve); *“contact points”* including who to go to for mental health and wellbeing support *(Steve)*; a “*checklist […] of what they need”* [e.g., equipment] *(Steve); “guidance on* [if any] *incidents”* are ongoing *(Steve);* and all *“necessary online training” (Erin).*

Having a support package was thought to also ease burden on managers: “*I felt very aware that I was super reliant on my manager who is trying on board you*,* but also working on other things […] I think it would have been really helpful if there was just a beautiful package which kind of lists everything you need to know” (Erin).* Some participants chose to share examples of similar workplace support packages that they have experience of. For example, one participant conveyed how they had made their own guidance for new recruits in their team (*“*[we] *did a sort of a welcome pack for all these students that we hired specific to the lab that they were working at” (Emma)*, as well as resources available in another organisation: “*We have a wellbeing package. That is an online thing*,* whether it is for financial*,* emotional*,* any kind of support*,* you can go to look at resources or training. Whether it’s from menopause*,* to sleeping*,* to if you’re in financial worries*,* anything at all […] which is amazing.” (Bethany).*

Two participants suggested exercising or trialling transitions to ways of working where possible to improve preparedness for future incidents. Participants believed this would provide the opportunity to flag more logistical considerations for employees: “*If you live in shared accommodation*,* is it even reasonable to expect people to work from home? and if it is not reasonable to expect them to work from home*,* what alternatives can we consider?” (Daniel)*, as well as allow for trialling of communication from organisations and standard operating procedures. Preparation depending on different contexts was also mentioned “*it depends on whether you envisage your situation of being a pandemic and where we shove everybody home because we have to or whether we’re looking to accommodate a sort of a humble normal working pattern” (Daniel).*

Many participants suggested bespoke training for line managers to help with adapting to being unable to see employees in the workplace: “*some people in my team had some problems and the only reason that people knew that they had problems was because they were in the office and that they could see them […] so something for managers that are managing people that are working from home in an incident […] to kind of go*,* these are the warning signs. (Olivia)*”. Nearly all training suggestions concern wellbeing, and one participant summarised the importance of “*keep[ing] well-being at the heart of everything they* [managers] *are doing*,* especially because roles in* [redacted] *particularly get extremely demanding and challenging” (Gemma).* Specific areas to cover in training included: how to handle challenging management situations in remote formats; how to keep a balance between “*keeping tabs*” and not being a “*micro manager*” *(Jack)*; how to provide opportunities for social interaction with others; how to identify when someone has “*bad habits” (Amy)*, for example not taking breaks or annual leave, or working over arranged hours; how to identify signs indicating that employees may be struggling; and, how to feel comfortable having wellbeing related conversations (e.g., “*soft skills” - Josh*).

### Experience of contributing to the response effort

The theme of experience of contributing to the response effort encompasses four sub-themes (privilege, workplace demands, external support, moving and taking breaks), and ends with participants suggestions for wellbeing support whilst responding to public health emergencies.

### Privilege

Some participants expressed contributing to the pandemic response was a privilege that provided *“meaning”* and *“purpose” (Emily)* for employees, as well as being an *“exciting”* and *“remarkable” (Jade)* experience: *“it’s been like the biggest privilege of my career to work on COVID […]*,* probably I have learned what normal people in the civil service learn in five years in one or two” (Jane)*.

### Workplace demands

The response-based work demands, and pace required, were tiring: *“I did find myself working quite long hours*,* you know*,* the work had to be stretched out over quite long hours*,* which in some ways was good that I was able to do that and work flexibly. I also was able to get overtime*,* which was good. But it was tiring at times” (Cara)*. Participants stated the work was fast paced, intense, and generally became busier with longer hours.

### External support

Support from colleagues, and those in the household (e.g., family and friends), seemed essential for some participants: *“It was tough. I did cry*,* and my husband pulled me away from my laptop and made me log off for half an hour and calm down. I was just frustrated*,* and I was tired*,* and it was tough. But I picked myself up and I carried on and that’s what you had to do” (Molly*). Those without much support found the working from home on the response more difficult: “*I found it very*,* very difficult to work from home*,* but I think it was also compounded with the fact that like I don’t have like family support nearby or anything like that. […] it felt like I didn’t have much of a network” (Grace)*.

### Moving and taking breaks

Some participants were concerned with the sedentary lifestyle associated with working from home on the response: *“the lack of movement. The weight gain*,* the lack of exercise” (Kim)*. As well as having low level concerns about taking breaks: “*You’ve gone yellow [reference to Microsoft Teams away feature]. Oh*,* my goodness. […] Somebody somewhere is judging me. Whereas if I were in the office it*,* you know*,* I could go to the bathroom and not worry about somebody’s wondering where I am. (Cara)*”.

### Participants suggestions for wellbeing support

Many participants provided suggestions of specific support for mental health and wellbeing, which included: checklists for simple healthy behaviours and behavioural nudges (e.g., “*have you brushed your teeth today? Have you showered? Are you looking after yourself?*” *(Lauren)*), tips and tricks for maintaining healthy behaviours (e.g., sleep hygiene: “*don’t sleep in the same room where you’re working*” *(Jade)*), guidance for mental health check-ins, advocating for existing mental health support, such as the Employee Assistance Program, documented experiences of those working on response-based work (e.g., blogs or podcasts of lessons learnt) due to being easier to read and *“more relatable” (Mary)*, sessions for encouraging movement, and fitness apps. Participants also suggested blocked out time for reflection and re-cooperation with colleagues. More direct wellbeing support was also suggested, such as workplace wellbeing interventions: *“You can’t out yoga the pandemic […] actual like cognitive behavioural therapy*,* type interventions that we can practice and can be supported to do so” (Lauren))*. It is necessary to note that supporting wellbeing was not supported by all participants, two did state that wellbeing is something that they do not wish to talk about, and often will not read emails concerning wellbeing opportunities: “*just not my thing […]* [I] *don’t talk about it.” (Jack).*

### Work life balance

The theme of work life balance encompasses three sub-themes (convenience, household calling, blurred boundaries), and ends with participants suggestions for ensuring positive work life balance.

### Convenience

Many participants perceived the convenience that accompanies working from home positively. For example, many appreciated the ability to tailor the working day to personal needs (e.g., childcare, taking breaks in the garden, having lunch with family, completing household chores): *“I mean for childcare reasons. It just made things with them a lot easier […] Rather than being an hour away from having to do school pickups or anything like that*,* we were around*” *(Olivia)*.

The lack of commute and rigorous start/end times, the lack of distractions whilst working from home, and the ability to work comfortably (both in terms of attire and workplace) were viewed very positively and aligned with decreased levels of financial outgoings (e.g., *“I wasn’t putting fuel in the car*,* wasn’t paying to park*,* wasn’t buying lunches” (Louise)*) and increased concentration and productivity: *“I think it was more productive and I was definitely very focused. I think working from home helps that because you didn’t have any distractions of people” (Jade)*.

### Household calling

Participants recalled having concerns about the impact working from home had on their home lives. Initially, being able to stop oneself from wanting to make use of the time at home (e.g., wanting to complete household chores) was difficult. Having others at home whilst trying to work was also cited as problematic, this was particularly true for those with young children needing to be home-schooled: *“my children had just started reception. So*,* it was probably some of the worst ages to try and home school a child*,* so that was difficult. Like I said*,* if it happened now*,* it would be very easy - they’re much more independent” (Cara)*, or just more generally in relation to having others at home whilst trying to work: *“I was living with my husband*,* and we had a flatmate as well and the cat […] so there were quite as quite a lot of other things happening in the periphery that I wouldn’t normally experience in an operation centre” (Lauren)*.

### Blurred boundaries

Many participants also reported blurred boundaries between work and home life, with many struggling to switch off from work: *“I think it disrupted home life a little bit more than expected*,* and actually I struggled to disconnect from work and […] the two blur into one […] not only being in the response and living it through work and at home*,* but also then not be able to disconnect because there was no disconnect” (Peter)*. Despite being challenging, many participants did report developing methods to maintain boundaries between work and home life over time: *“I think with working from home*,* if you if you don’t put in sort of structure*,* it’s very easy to just work*,* finish work*, [then] *you’re asleep kind of situation” (Amy)*. For example, some followed an office-like routine, such as going into a designated office space or going for a walk before or after work. Other methods included: trying to remove focus from COVID-19 after work (e.g., “*listening to a podcast or try and do something that takes my focus away from COVID” (Harry))* and using those in the household to help keep a separation between work and home life (e.g., *“I can hear my partner’s getting up*,* we’re going to go cook dinner together” (Erin)*).

### Participants suggestions for aiding positive work life balance

Participants expressed the importance of setting expectations when working from home. This exercise was suggested, for example, between line managers and employees (e.g., *“have an upfront conversation about it. OK*,* I’m gonna [sic] switch my out of office on at 5:00 every evening. And don’t expect a response until the next morning” (Jack))*, as well as having blanket guidelines for all (e.g., *“expectations are that*,* you know*,* even though it’s an enhanced incident*,* you should still be working sensible hours and not overdoing it” (Louise)*). Akin to this, one participant shared an example of ‘expectation setting’ to show what a good routine looks like: *“One thing [X] did*,* who was my manager at the time*,* he put together this dream day. He’s like if we’re doing a good job today on the response like it will look like you logging on at this time*,* you’ll take a break every hour for 5 minutes […] you’ll take time away from your desk…” (Lauren)*. Setting expectations would also alleviate any concerns employees may have about taking breaks whilst working from home.

### Relationships with colleagues

Being separated from colleagues while trying to maintain active and open communication was difficult for many participants: *“from a professional point of view*,* you sort of lost the art of having a conversation across a desk. You’d have to call someone on Teams […] and they might not be there*,* they might be another call*,* and that’s tricky” (Harry)*. Not having the opportunity to meet in person was also referenced as an issue, particularly for new starters. Making genuine friendships was also a concern: *“it’s harder to make genuine friendships when you’re working from home” (Helen)*. Participants also suggested that being separate from colleagues began to be more difficult over time: *“the longer it was going on*,* […] you did start to miss casual interactions*,* you know*,* cracking a joke and things like that because everything was serious*,* and everything was meeting orientated. With time*,* you know I didn’t notice at the beginning” (Jade).*

### Participants suggestions for maintaining social connectedness

*Check-ins*: To maintain connections between colleagues, participants advocated for regular check-ins and up to date dairies/calendars for availability: *“I can see they’ve got nothing on between 2:30 and 3:00. So*,* I can give them a call then and hopefully they’ll be around” (Harry).* Maintaining lots of different communication channels and having software that provided opportunities for face-to-face interaction was also seen as advantageous.

*Maintaining contact*: Many participants expressed the importance of creating and maintaining team communication and regular contact, essentially *“making sure people are not isolated and alone” (Heather)* by manufacturing “*water cooler moments” (Daniel)* and providing opportunities for these to take place: “*having spaces where I can connect with people in a more informal way and I think that helps like during incidents response*,* because it gives you that kind of relief from the intensity of the work that that you’re doing” (Grace)*. Participants appreciated that maintaining and building these connections requires time and effort, but was also thought to provide opportunities to break down barriers between staff: “*if you’re just talking from like a junior position*,* I suppose*, [and] *having to like support people who are very senior*,* having […] just like a little catch up maybe*,* so you have that connection with the senior staff as well” (Grace).* Ensuring opportunities to divulge about working constraints (e.g., due to childcare, family illness, emergencies) was also mentioned by participants.

*New starters*: A focus on involving new starters to ensure they are well integrated and well supported in new virtual teams was mentioned by many participants. For example: *“we had inductions for all our staff*,* and everyone was like booked in to have a one-on-one chat with people” (Jack)*. Buddy schemes (e.g., being paired with an established employee, or another new starter, to share your experience with) were also mentioned by participants.

*Relationships with managers*: Making active efforts to maintain a positive and engaged relationship between line managers and employees was deemed important by participants: *“engagement with your line manager is important because you can. And talk to them on a*,* you know*,* like a one-to-one level about things that are bothering you “(Jade)*. Indeed, participants stated the importance of being, or having, a contactable line manager: “*the best thing that I can do is just ensure that the person that I line manage know that they can contact me at any time. You know*,* my door is always open.” (Cara).* Furthermore, having a manager willing to speak about more personal areas (i.e., not just work-related conversations), such as “*being open themselves about what they are kind of struggling with” (Jane)*, and leading by example, (essentially role modelling good behaviour), was viewed positively by participants.

*Tailored support from managers: C*onversing with employees about preferred management style was suggested (e.g., *“line managers asking the person they’re supporting how often do you want one-to-ones?” (Grace)*, as was discussing frequency and scheduling of contact (e.g., *“either have a suggested template or something that they can modify just to start that contact with people and support whilst working from home” (Olivia)*). It was suggested these conversations should be carried out rapidly during the initial stages of homeworking: “*I think having the conversation up front. Rather than letting just people work it out themselves. (Jack)”.*

### Space and equipment

Many participants felt underprepared for working from home, and some were missing the practical equipment they needed (e.g., desks, chairs) and lacking in IT support to work effectively: *“I set up like a temporary desk*,* basically on an ironing board” (Jade)*. Similarly, some participants simply did not have the space needed to work from home: *“I was living alone at that point and I had one room which functioned as living room*,* eating room*,* working room and literally my whole everyday room where I worked all day for many more hours than is normal at the time*,* due to the situation*,* and didn’t have any way of getting away from that” (Mary)*.

### Participants suggestions for space and equipment support

Many participants stressed the importance of having the correct equipment needed to work from home comfortably and effectively: *“Number 1*,* Equipment*,* like making sure you have like the right equipment to be able to work comfortably at home” (Helen).* Suggestions included having an IT kit ready to go (e.g., *“that includes a desk*,* chair*,* two monitors and a decent laptop”* (Molly)) and instructions of how to set it up ergonomically (e.g., *“in a way that’s not going to hurt your back”* (Grace)).

Participants believed having a robust IT system, IT helpdesk, and additional IT resources are required. For example, *“a high-level manual*,* that would have really helped. I definitely spent a lot of the time in my first few weeks on the phone to*,* or on hold to*,* IT going where does this cord go? and what’s Teams? They could have saved themselves a lot of time with some basic IT support (Lauren)”*. Additionally, participants suggested training on workplace software would be useful when transitioning to working from home: *“Maybe ensuring that people know how to use things like Teams [Microsoft Teams] properly. I think at first that was something we all struggled with a little. (Cara).*

### Inclusivity

For some, working from home provided the opportunity to be able to work, despite many employment concerns during the pandemic (i.e., furlough). Additionally, many participants appreciated the safety they felt whilst working from home, essentially due to not mixing with others in the workplace: *“at the time we were also shielding as well*,* so it made it easier for us to do that” (Gemma).* Working from home as a concept was also considered more inclusive for those with anxiety, and those with additional needs (e.g., due to injury or illness, due to undergoing treatment): *“the trouble with the treatment I was going through*,* I was knackered*,* so I would quite often sleep during the day and then wake up at three in the morning thinking I’ll do some work and so I found easy the flexibility of the work was good” (Emma).*

### Ways of working preference

Nearly all participants expressed a preference for future hybrid working (e.g., a combination of both office and home-based work), with many believing that this arrangement allowed them to have *“the best of both worlds” (Jane)* and be more flexible. In relation to going into the office, participants liked the opportunity for social interaction with colleagues *(“it’s good to be able to have meetings with people face to face” (Sue)*), being able to use office-based equipment (“*I think from a practical point of view*,* access to a printer” (Louise)*, and have the opportunity to be more active with a change of scene: *“Also being able to move to out this chair*,* walk a bit*,* pop to town*,* see the community*,* drive the car” (Kim)*. In terms of working from home, participants appreciated the flexibility *“lifestyles have changed*,* you know*,* around families and things or school things” (Amy)*, having a quiet environment to work in *“it’s quiet so it’s easier to concentrate”* (Sue), and well as being able to choose what your day looks like *“I feel like in the office it’s like managed by kind of what everyone else is doing as well* [be]*cause everyone’s talking or that everyone’s going to lunch at specific time and you don’t want to like be left behind” (Helen).*

### Participants suggestions for supporting workplace changes

A few participants stated the importance of not only providing support and guidance for the transition to working from home, but for the shift back to working in an office. For example: “*maybe not enough focus is being put on the people that actually now they’ve had a bit of time away*,* might find it hard to go back* [to the office].*” (Cara).*

## Discussion

This qualitative study had two main aims. First, to explore frontline UK Government employee’s experiences of responding to the COVID-19 pandemic whilst working from home and second, to establish what support and guidance employees were offered. Our findings suggests that, during the pandemic, civil servants working from home reported feeling a strong sense of purpose and achievement. However, they also found the work was extremely demanding, particularly for those who had to rapidly, and unexpectedly, transitioned from office or lab work to home working. More generally, the nature of their homeworking experience depended on a range of practical (e.g., equipment and space), organisational (e.g., relationships with managers) and personal factors (e.g., caring responsibilities, living situations). Furthermore, participants provided a wealth of information of relevance for future support provisions for frontline UK civil servants working from home whilst responding to public health emergencies.

Participants reported that their experiences of working from home on the pandemic response were mixed with very few regarding the experience to be wholly positive or negative. This finding aligns current literature, as working from home was found to have mixed impacts on mental health and productivity [6], along with multiple factors that can influence perception of homeworking [7]. However, nearly all participants expressed a preference for hybrid working in the future. This preference has also been reported in Foreign, Commonwealth & Development Office employees [24]. In the current study, participants preferred hybrid working as it allowed them to maximise benefits of homeworking (e.g., flexibility) and overcome difficulties (e.g., seeing colleagues). Should UK response-focused civil servants be asked to work solely from home on incident response again in the future, they should be provided tailored support which accounts for varying personal contexts and individual differences.

Most, but not all, participants were able to recall various support offers being provided by the organisation. However, the nature of the support offered to participants varied considerably, suggesting that better advertising of support offers is required. As a result, it is recommended that employers consider developing an accessible repository of all available support offers. This may help streamline the process of employees reaching out for support if needed. How to access the repository could form part of the induction for all new joiners, to aid accessibility. Additionally, published literature suggests that individuals are more likely and willing to seek mental health support if aware, or connected, with others who have sought help themselves [25, 26]. Therefore, the use of promotional videos, or talks, by people who have used offered support and found it helpful are also recommended.

The attitudes of line managers highly influenced participants’ perceptions of working from home. Participants generally appreciated open and honest management and enjoyed being able to converse with managers about non-work-related information. Research suggests an association between poor management style and mental ill-health [27]. Which showcases the importance of managers being adequately educated, in not only management style, but how to support employees effectively. Working from home as a manager may require greater forethought and adaptation of management style (i.e., how to manage employees when you can no longer see them). As a result, it is recommended that managers should receive training in adapting their usual management style to suit working from home, as well as being trained in ‘soft skills’ to allow for regular competent wellbeing check-ins with employees.

Social connections, working relationships, and support from outside of the working context (e.g., from friends and family) all played a role on participants experience of working from home. Namely, others in the household often aided in setting boundaries between the workplace and home, as well as creating a comfortable environment for employees whilst working in a stressful context (i.e., on public health emergency response). One participant explicitly attributed having a worse experience of working from home on the response due to not having ample social support. These findings align with research which suggests homeworking employees may lose the ability to create a shared sense of social identity with colleagues [28], which is protective of workplace stress [29] and burnout [30]. Therefore, employers and managers should be proactive in allowing teams to build in resilience, which requires positively facilitating activities and communication between team members (e.g., informal catchups, and opportunities to work collaboratively).

In terms of preparedness during the initial move to working from home, unsurprisingly those working from offices at the time of the announcement were not well prepared to make the transition. However, this study demonstrates that response work can be run from employees’ homes and is manageable for many. However, more rigorous protocols are recommended: organisational preparedness could come in the form of exercising or trialling future potential changes to ways of working. This would allow for preparation of communication with employees as well as allow for practical considerations to be raised (e.g., how to support employee X if they have no desk at home; how new starters will be integrated into teams). Similarly, participants expressed needing to be prepared, and having support, when other changes to ways of working are implemented (e.g., during a move back to office working). Changes should seek to be as inclusive as possible, as well as ideally providing time for employees to adjust accordingly.

Our results also suggest that employees should be provided with appropriate equipment to enable home working comfortably and safely, where it is economically viable to do so. Managers should also encourage staff to regularly review their needs should they change. Furthermore, employees should be trained, or have otherwise developed competency in, the organisational communication software packages which would optimise the connections between employees, as well as increase efficiency. Lasty, due to varied contexts and situations in the homes of those working, tailored training on how to create separation between work and home life (e.g., blurred boundaries, distractions at home, help with setting expectations) should be available to employees.

### Implications

Learning from organisational crises (in this case, the COVID-19 pandemic) is imperative for organisational preparedness for future crisis events [31]. Due to the likelihood of future infectious disease outbreaks, along with various other public health emergency contexts which may require working from home, it remains important for UK Government Organisations to easily pivot how and where response-focused civil servants work to facilitate an effective response effort. In the case of the UK Civil Service, turnover of employees is high (i.e., civil servants moving to different departments, or leaving the Civil Service entirely; [32]), which reduces institutional memory [32]. Taking this into account, civil servants that experienced working on the COVID-19 response from their own homes are unlikely to work on the next public health emergency requiring homeworking. This results in newer civil servants encountering the same challenges to overcome (as their predecessors have previously experienced) in future similar contexts. This highlights the importance of drawing and learning from employee experience during the COVID-19 pandemic, such as done in the current study. Therefore, based on the findings highlighted in this study, we have made a series of recommendations for facilitating effective homeworking in future emergencies which can be found in Table 2. For ease, the recommendations have been segregated into those relevant for employers/organisations and managers.

**Table 2:** Recommendations for employers, organisations and managers to undertake when response-focused employees are asked to work from home during future public health emergencies

	Recommendation
Employers / Organisations	A repository of available and accessible support should be created and advertised.
	Appropriate support should be provided when adapting to any alteration in ways of working (e.g., moving back to the office).
	All staff should be provided with necessary training for all communication software used by the organisation.
	Employers should be proactive in allowing teams to build in resilience, which requires positively facilitating activities and communication between team members.
	Home working equipment, which is both safe and comfortable, should be provided by employers where possible.
	New starter inductions should include information about the organisation (e.g., strategy, aims, purpose) as well as advertising organisational support mechanisms.
	Employers should provide tailored support (considering individual differences and personal contexts) when asking employees to work from home.
	Organisational preparedness could come in the form of exercising or trialling future potential changes to ways of working before they happen.
Managers	Employees should be encouraged to access support through the use of videos/presentations or similar outreach methods in a way that makes accessing support appear routine and beneficial.
	Managers should be trained in ‘soft skills’ to allow for regular competent wellbeing check-ins.
	Managers should have training on adapting their management style to suit working from home.
	Managers should be proactive in allowing teams to build in resilience, which requires positively facilitating activities and communication between team members.
	Managers should provide tailored support (considering individual differences and personal contexts) when asking employees to work from home.

Logically, the next steps of this research would be to refine these recommendations, and ensure they are tailored and actionable for response-focused civil servants. In order to do this, a follow-on Delphi study (i.e., a technique used to systematically combine expert opinion in order to arrive at an informed group consensus on a complex problem [33]) has been employed by the authors of this article [34].

In relation to wider research implications, this exploration into experiences of UK response-focused civil servants supports the use of an occupational-specific approach when investigating working from home. In essence, due to the mixed nature associated with working from home (i.e., due to varying contextual and situational factors), examining experience on a case-by-case basis (in relation to occupation) allows for in-depth exploration to detail key barriers and facilitators experienced by employees. This provides the opportunity for employers/managers to target areas for improvement during future similar events.

As working from home is now a practical solution to reducing infectious disease transmission [35] and may also be useful during other public health emergencies (e.g., infectious diseases, natural disasters, chemical and radiological incidents), exploration into other populations using similar methods as followed here, would be beneficial. This would also allow for comparisons within and between different occupations.

### Limitations

Despite being the first paper to examine the experience of working from home in this population, it is not without limitations. Firstly, one author coded and analysed the data. However, the emergent data was often discussed in research team meetings, as well themes being iteratively developed based on team feedback. Secondly, the sample size is relatively small (at 30 participants), but each interview lasted around 40 minutes providing a wealth of data [18]). Thirdly, Civil Service statistics report that within the Government Organisation at hand, around 65% of employees in 2023 were female [36]. Therefore, it is important to note that 80% of the sample within this study were female, providing a slightly inflated rate in comparison. This is important to note given the potential divergence in viewpoint on remote work between genders, influenced by familial roles (e.g., additional papers for interest in this area can be found here [37–39]. Akin to this, participants were not asked for their marital status within this study which may have provided an avenue for segregating findings. However, the number of people residing within the household participants were working from was collected, and findings related to this can be pinpointed in the theme of ‘*Work life balance*’. Additionally, it is possible that those with strong feelings relating to working from home were more likely to volunteer themselves to take part in the study (e.g., for example, two participants were relieved to work from home due to chronic conditions and mobility issues); any relevant contextual information that could aid in interpretation of the results has been provided in the results section. Lastly, given that interviews were conducted between August and September of 2023, it is important to consider participants’ ability to recall details from their initial transitions to working from home during 2020 may be hindered due to the amount of time passed since the event. Essentially, more timely research may have potentially provided additional insights. However, given the importance of reflection and drawing on employee experience to improve future organisational practice, the findings from the current paper remain important.

## Conclusions

This interview study collected and analysed data from 30 UK Government response-focused employees who responded to the COVID-19 pandemic from their own homes. A total of eight themes were derived from the data. In summary, participants described their overall experience of working from home to be mixed, and differed due to various contextual and situational factors (e.g., having children at home, living in a confined space). However, the vast majority favoured a future pattern of hybrid working. Many participants were underprepared to work from home, but results suggest that there are a number of ways that organisations which employ emergency response staff can better prepare and support them should they have to work from home again during future public health emergency and response. However, further work is required to ensure these recommendations can be implemented by relevant stakeholders and they require evaluating to ensure they are fit for purpose and effective for use during any future public health emergency.

## Electronic supplementary material

Below is the link to the electronic supplementary material.

Supplementary Material 1

Supplementary Material 2

Supplementary Material 3

Supplementary Material 4

## Data Availability

Data cannot be shared publicly because of ethical restrictions: participants were guaranteed in their Study Information Sheets that their interviews would not be publicly available. As the data contains potentially identifying or sensitive information about governmental staff the raw data is accessible only by members of the research team.
