# Supplementary material for: “We’re all in the same storm, but not all of us are in the same boat”: qualitative exploration of UK response-focused civil servants experiences of working from home during COVID-19
Source: BMC Public Health. 2025 Jan 23;25:289. doi: 10.1186/s12889-025-21385-4 (PMC11761760; doi:10.1186/s12889-025-21385-4)
Supplement: Supplementary file 1 — Supplementary Material 1 [file 12889_2025_21385_MOESM1_ESM.docx]

**Interview Guide**

***Transition to homeworking***

- Today we are interested in firstly gathering your perceptions of your initial transition to homeworking in [REDACTED]
- Option 1: So firstly, if you could take yourself back to March/April of 2020 when the UK government asked the public to ‘work from home if you can’ due to the virus.

1. Please could you tell me how you first found out that you would be working from home?

- What were your initial thoughts about this?
- How did the news make you feel?
- Could it have been delivered differently, if so, how?
- Option 2: So firstly, if you could take yourself back to when you joined the organisation and you began working from home on the COVID-19 response.

1. How did you feel to be starting a new role whilst working from home?

- What were your initial thoughts about this?
- How did it make you feel?

1. Could you tell me about your initial transition to homeworking during the pandemic?

- What was your experience of the transition like?
- What were some common thoughts or feelings you had about working from home?
- Can you tell me about something you initially liked about homeworking?
- Can you tell me about something you initially disliked about homeworking?
- Did you feel prepared to work from home? Why?

1. Initially, did working from home impact your ability to work?

- Can you tell me anything that initially made it easier/ for you to work from home?
- Can you tell me anything that initially made it harder for you to work from home?
- Anything else?

1. Were you provided with any support when you were initially working from home [e.g., guidance documents, toolkits, line manager meetings]?

- If yes, what were they? were they beneficial?
- If no, what do you think would have been beneficial to you?

***Getting used to Homeworking during COVID***

- These questions are related to your experiences after working from home on the COVID response after a few months.

1. How did you find working from home during COVID after a few months had passed?

- What were some key thoughts and feelings you experienced?
- Can you tell me about any changes you noticed (e.g., in working routine or lifestyle)?
- Could you tell me any changes you experienced in your job role due to COVID over this time?

1. Could you tell me about your experience after a few months of homeworking in comparison to the initial period?

- What were you feeling and thinking?
- Can you tell me about anything you liked about homeworking after a few months?
- Can you tell me about anything you disliked about homeworking after a few months?
- Did you feel any more prepared to work from home? Why?

1. At this stage (after a few months) had you been provided any support whilst working from home [e.g., guidance documents, toolkits, line manager meetings]?

***Reflection***

- These questions are related to lessons learnt whilst working during the pandemic, and reflection on your experiences.

1. Could you briefly summarise your experience of working during the pandemic?

- Were there any bits particularly easy/difficult for you and why

1. Were there any changes, or did you make any changes, that made it easier or harder for you to work from home?

- Organisational (e.g., changes in structure, job role)
- Workload (e.g., COVID demands, deadlines)
- Personal (e.g., childcare, logistics of home working)

1. Could you tell me some key things you have learnt from homeworking during covid?

- What would you consider to be the main barrier/difficulty for you working from home? Do you have any suggestions on how to overcome X barrier?
- What would you consider to be the main facilitator for you working from home?

1. What would be your perfect way of working in the future? Why?
2. What advice would you give to someone working from home during a public health emergency whilst responding to an enhanced incident?

***Support packages (10 minutes)***

- This research aims to create some form of support package for those rapidly transitioning to homeworking in the future whilst working on an enhanced response.

1. Based on your experiences of working from home, what would you like to see or what would you find useful in support packages for people transitioning to homeworking in the future?

- Format?
- Information to include?
- Who should they be aimed towards? (e.g., management vs employee vs organisation)
- What would make you feel supported?
- How could you support others?

***Final remarks***

1. That brings us to the end of the interview.
   - Is there anything else you would like to add on how homeworking during COVID-19 impacted you?
   - Are there any positive or negative factors, or barriers and facilitators, that you encountered that you would like to talk about?
   - Are there any final conclusions on what would make homeworking during a public health incident easier for you?
