# Supplementary material for: “We’re all in the same storm, but not all of us are in the same boat”: qualitative exploration of UK response-focused civil servants experiences of working from home during COVID-19
Source: BMC Public Health. 2025 Jan 23;25:289. doi: 10.1186/s12889-025-21385-4 (PMC11761760; doi:10.1186/s12889-025-21385-4)
Supplement: Supplementary file 2 — Supplementary Material 2 [file 12889_2025_21385_MOESM2_ESM.docx]

**Study summary for participants**

**Distributed by gatekeepers:**

We are looking for [redacted] employees who worked on the COVID-19 response whilst working from home. Discussions will be focused on your experiences of working from home whilst responding to the COVID-19 pandemic. The interview will take up to an hour and you will be compensated with a £25 Love to Shop voucher for your time.

Any data you provide will remain confidential and will feed into the development of materials to aid the transition to homeworking for current and future response staff. If you are interested in taking part, please register using the following link:
