# Supplementary material for: “We’re all in the same storm, but not all of us are in the same boat”: qualitative exploration of UK response-focused civil servants experiences of working from home during COVID-19
Source: BMC Public Health. 2025 Jan 23;25:289. doi: 10.1186/s12889-025-21385-4 (PMC11761760; doi:10.1186/s12889-025-21385-4)
Supplement: Supplementary file 3 — Supplementary Material 3 [file 12889_2025_21385_MOESM3_ESM.docx]

Expression of Interest - Interviews

**Working from home during COVID-19: Interview study**
 
*Please note, we will be conducting a total of 30 interviews. If you are chosen to participate, one of the research team will be in contact shortly via the email address you provide at the end of this short screening questionnaire.*

Do you have experience of working for [REDACTED] from home during the COVID-19 pandemic?

- Yes (1)
- No (2)

Did you contribute to the COVID-19 response whilst working from home?
*(e.g., secondment, took up additional duties, BAU work became pandemic related, new role, etc.)*

- Yes (1)
- No (2)

Which of the following areas do you currently work in? (**Click directly on image to choose areas**, if applicable, please select multiple options)

[Image of organisational structure]

If other, please indicate here:

*E.g.,* ***for those who no longer work for [REDACTED]***

During the pandemic, which of the following areas did you work in? (**Click directly on image to choose areas**, if applicable, please select multiple options)

[Image of organisational structure during COVID]

Please describe other:

________________________________________________________________

Please enter your email address below:

________________________________________________________________

________________________________________________________________
